# Equol Nonproducing Status as an Independent Risk Factor for Acute Cardioembolic Stroke and Poor Functional Outcome

**DOI:** 10.3390/nu16193377

**Published:** 2024-10-04

**Authors:** Kotaro Noda, Yorito Hattori, Hiroaki Murata, Yoshihiro Kokubo, Aya Higashiyama, Masafumi Ihara

**Affiliations:** 1 Department of Neurology, National Cerebral and Cardiovascular Center, 6-1, Kishibe-shimmachi, Suita 564-8565, Japan; 2 Department of Neurology and Neurological Science, Graduate School of Medical and Dental Sciences, Institute of Science Tokyo, 1-5-45 Yushima, Bunkyo-ku, Tokyo 113-8519, Japan; 3 Department of Neurology, Department of Preemptive Medicine for Dementia, National Cerebral and Cardiovascular Center, 6-1, Kishibe-shimmachi, Suita 564-8565, Japan; 4 Department of Preventive Cardiology, National Cerebral and Cardiovascular Center, 6-1, Kishibe-shimmachi, Suita 564-8565, Japan

**Keywords:** equol, stroke, cerebrovascular disease, cardioembolic stroke, atrial fibrillation

## Abstract

**Background/Objectives**: Equol has protective effects against coronary artery disease and dementia by strongly binding to estrogen receptor beta, whereas the intake of soy isoflavone alone does not always confer such protective effects. Equol production is completely dependent on the existence of equol-producing gut microbiota. The effects of equol-producing status on the cerebrovascular diseases remain unclear. The current study was aimed to investigate the association of equol-producing status with the development of stroke and its neurological prognosis. **Methods**: Frequencies of equol producers were compared between healthy subjects (HS) registered in the Suita Study and patients with acute stroke admitted to our stroke center from September 2019 to October 2021 in a retrospective cohort study. **Results**: The proportion of HSs and patients with ischemic stroke who were equol producers did not significantly differ (50/103 [48.5%] vs. 60/140 [42.9%], *p* = 0.38). However, cardioembolic stroke was significantly associated with low a prevalence of equol producers (adjusted odds ratio [aOR] 0.46, 95% confidence interval [CI] 0.21–0.99, *p* = 0.05). A higher left atrial volume index was observed in equol nonproducers (46.3 ± 23.8 vs. 36.0 ± 11.6 mL/m^2^, *p* = 0.06). The equol nonproducers had a significantly higher prevalence of atrial fibrillation than the equol producers (27.5% vs. 13.3%, *p* = 0.04). Furthermore, the equol producers exhibited a significantly favorable functional outcome upon discharge (aOR 2.84, 95% CI 1.20–6.75, *p* = 0.02). **Conclusions**: Equol is a promising candidate for interventions aiming to reduce the risk of CES and atrial dysfunction, such as atrial fibrillation and improve neurological prognosis after ischemic stroke.

## 1. Introduction

Soy isoflavones are present in soybeans and soybean products [1], and these substances comprise a subgroup of phytoestrogens. Further, they have estrogen-like properties due to their structural and chemical similarity to estradiol [2,3]. Notably, they bind to estrogen receptor beta, which is mainly located in the brain and systemic vascular system [4]. In vivo and in vitro preclinical studies have revealed that soy isoflavones have anti-atherosclerotic activity [5], antioxidant [6], and anti-amyloidogenic effects [7]. Clinically, a high soy isoflavone intake is associated with a reduced risk of cerebrocardiovascular diseases, particularly in Japanese [8] and Chinese [9] women. On the contrary, soy isoflavone supplementation does not suppress carotid artery intima-media thickness progression in white, black, and Hispanic postmenopausal women; however, Asian women are at lower risk for carotid artery intima-media thickness progression [10]. Thus, the beneficial effects induced by soy isoflavone may vary among races. Presumably, the abovementioned discrepancy can be explained by differences in equol-producing capacity between East Asians and Westerners.

Equol is metabolically transformed from the isoflavone daidzein by the gut microbiome. Its production is based on the presence of specific equol-producing microbiota, such as *Bifidobacterium* [11]. The proportion of equol producers varies based on dietary patterns, race, and ethnicity [12,13,14]. Approximately 50–70% of adult Asians produce equol, whereas only 20–30% of Westerners [15]. Equol had a significantly higher affinity to estrogen receptor beta than its precursor, daidzein and genistein, another major dietary source of soy isoflavone [16]. Previous studies have shown that equol has protective effects against coronary artery disease (CAD), with its urinary concentration having an inverse correlation with CAD incidence [17]. Additionally, a cross-sectional study has shown that a lower prevalence of coronary artery calcification is associated with a higher serum equol level [18].

Despite these findings, data regarding the effects of equol against cerebrovascular diseases are limited. Several in vivo studies have revealed that equol has neuroprotective effects in rodent models of focal cerebral ischemia [19,20,21]. Equol-producing status is significantly associated with a smaller volume of white matter lesions on brain magnetic resonance imaging (MRI) in older Japanese people with a normal cognitive function in a previous longitudinal observational study [22]. Regarding acute stroke, only one small-scale observational study has revealed that patients with stroke (*n* = 12) have a significantly lower abundance of *Adlercreutzia equolifaciens*, an equol-producing gut microbiota, than healthy controls (*n* = 30) [23]. However, its impact on acute stroke remains unclear. Therefore, the current study was aimed to examine the proportion of equol producers between patients with various acute stroke subtypes and healthy subjects (HSs). Moreover, the association of equol production status with neurological prognosis and cardio-cerebral imaging abnormalities across all-cause stroke cases was investigated.

## 2. Materials and Methods

### 2.1. Study Design

This retrospective cohort study was performed at the National Cerebral and Cardiovascular Center (NCVC), Japan. The study was conducted in accordance with the Declaration of Helsinki and was approved by the Research Ethics Committee of NCVC (approval numbers: R21044 and M27-066). We collected the data of patients with stroke and HSs from the electronic medical record. The proportion of equol producers was compared, and the association between equol-producing status and stroke subtypes and prognosis was investigated.

### 2.2. HSs

Healthy participants who were registered in the Suita Study, a population-based prospective-cohort study, were examined [24]. Participants aged 75–89 years who met our screening criteria (without a previous history of stroke, neurological disorders, depression under treatment, or other conditions) were randomly selected from the cohort of the Suita study between November 2016 and September 2018. All participants provided written informed consent. Clinical data including age, sex, body mass index (BMI), medical history, and laboratory results were obtained.

### 2.3. Patients with Stroke

The inclusion criteria were as follows: patients who were hospitalized due to acute ischemic stroke, including small-vessel occlusion (SVO), large-artery atherosclerosis (LAA), cardioembolic stroke (CES), and embolic stroke of undetermined sources (ESUS), and hemorrhagic strokes such as intracerebral hemorrhage (ICH) between September 2019 and October 2021, those who provided a written informed consent for the NCVC Biobank, and those with fasting blood samples collected within six months of stroke onset. Stroke subtypes were diagnosed based on the TOAST classification [25]. The exclusion criteria were as follows: patients with any type of cancers, those with serum samples collected during fasting for >24 h or when they took probiotic drugs, antibiotic drugs, immune-suppressive agents, or pioglitazone; and those without blood samples within six months of stroke onset. Notably, the amount of endogenous equol is preserved for at least 24 h from equol intake [26], and the equol-producing state is stable for at least 12 months [27].

After patient selection, the clinical data of the patients, including age, sex, BMI, medical history, functional outcome, CHA_2_DS_2_-VASc (congestive heart failure, hypertension, age ≥75 years, diabetes mellitus [DM], previous stroke, vascular disease, age 65–74 years, sex category) score [28], laboratory data, and transthoracic echocardiography (TTE) and brain MRI findings, were collected. The National Institutes of Health Stroke Scale (NIHSS) scores upon admission were assessed by well-trained vascular neurologists at our department.

### 2.4. Evaluation via TTE

TTE was performed at the NCVC. We analyzed left ventricle ejection fraction (LVEF), left ventricle diastolic diameter (LVDD), left ventricle systolic diameter, left atrial diameter (LAD), and left atrial volume index (LAVI). LVEF was visually estimated from the apical two- and four-chamber views or was measured using the Simpson’s or Teichholz method. LAD was measured on the parasternal long-axis view. LAVI was evaluated by dividing the LV volume estimated from the apical four-chamber views based on body surface area.

### 2.5. Evaluation of Cerebral Small Vessel Disease

We assessed the neuroradiological findings of cerebral small vessel disease (SVD) including lacunes, cerebral microbleeds (CMBs), and white matter hyperintensities such as periventricular hyperintensities (PVH) as well as deep and subcortical white matter hyperintensities (DSWMH). Lacunes, CMBs, and white matter hyperintensities, as depicted in a previous study, were identified [29]. The presence of lacunes, CMBs, PVH, and DSWMH was evaluated independently by well-trained neurologists, and PVH and DSWMH were graded with a scale of 0–3 using the Fazekas scale [30]. After the evaluation, the total SVD severity score (range: 0–4), as described in a previous study, was calculated [31]. The presence of grade 3 PVH, grade 2 or 3 DSWMH, ≥1 lacunes, or ≥1 CMBs was each assigned with a score of 1, and the sum of these points was used as the total SVD severity score (range: 0–4). Grade two or three DSWMH was defined as overt DSWMH [32]. A total SVD severity of ≥2 was defined as severe SVD [33].

### 2.6. Serum Equol Concentration

Serum samples collected from the patients were stored in the NCVC Biobank. The serum equol concentration was measured via liquid chromatography-mass spectrometry (Kyusyu Pro Search LLP, Fukuoka, Japan). Equol-producing status was defined as an equol concentration of ≥1.0 ng/mL [34,35,36,37,38].

### 2.7. Statistical Analysis

Data were presented as mean (±standard deviations) for continuous variables and as percentages for categorical variables. The Fisher’s exact test or the Mann–Whitney *U* test was used to evaluate differences in categorical or ordinal variables. The Mann–Whitney *U* test or the *t*-test was utilized to evaluate differences in continuous variables between groups, as appropriate. Bonferroni correction was applied after one-way analysis of variance for multiple comparisons of categorical variables to identify differences between HS and patients with stroke. A modified Rankin scale (mRS) score of 0–2 upon discharge was defined as favorable functional outcome [39]. To identify the significant predictors of favorable functional outcomes, univariate and multivariable logistic regression analyzes were conducted. The multivariable logistic regression analysis was adjusted for age, sex, hypertension, diabetes mellitus, and dyslipidemia. Odds ratios (ORs) with 95% confidence intervals (CIs) were calculated. HSs and patients with ischemic stroke were compared as a primary analysis. HSs and patients with ICH were compared as a supplemental analysis. All reported *p* values were two-tailed, and *p* values of <0.05 were considered statistically significant. All analyzes were performed using the Statistical Package for the Social Sciences software version 27 (IBM, Armonk, NY, USA).

## 3. Results

### 3.1. Baseline Characteristics of HS and Patients with Ischemic Stroke

We enrolled 103 HS and 140 patients with acute ischemic stroke who were admitted to our hospital from September 2019 to October 2021. The mean ages of HS and patients with acute ischemic stroke were 81.3 ± 3.3 and 71.2 ± 13.6 years, respectively (*p* < 0.01). The proportions of male HS and patients with stroke were 50.5% and 72.9%, respectively (*p* < 0.01). Furthermore, the proportion of patients with acute ischemic stroke who had medical history such as hypertension and atrial fibrillation (AF) was significantly higher than that of HSs (54.4% vs. 78.6%, *p* < 0.01; 2.9% vs. 21.4%, *p* < 0.01) (Table 1). Among 101 patients with ischemic stroke who were treated with low-density lipoprotein cholesterol (LDL-C)-lowering drugs, 30 (29.7%) did not exhibit dyslipidemia upon admission. Meanwhile, 71 (70.3%) patients were still presented with dyslipidemia despite treatment with LDL-C-lowering drugs (Table S1).

### 3.2. CES and AF Were Associated with Equol Nonproducing Status

**Figure 1:**
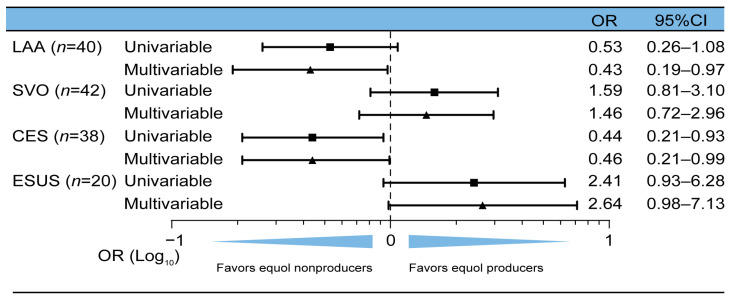
Logistic regression analyses of equol-producing status associated with stroke subtypes. Logistic regression analyses showed that cardioembolic stroke (CES) was significantly associated with equol nonproduction. Multivariable logistic regression analysis was adjusted for age, sex, diabetes, dyslipidemia, and hypertension. Abbreviations: LAA, large-artery atherosclerosis; SVO, small-vessel occlusion; ESUS, embolic stroke of undetermined source; OR, odd ratio; CI, confidence interval.

The proportion of HS and patients with various ischemic stroke subtypes who were equol producers was compared. The proportion of HS and patients with ischemic stroke who were equol producers was comparable (50/103 [48.5%] vs. 60/140 [42.9%], *p* = 0.38). Of 243 participants, 110 (45.3%) were equol producers. The proportion of male equol producers was significantly higher than that of female equol producers (70.9% vs. 57.1%, *p* = 0.03) (Table S2). The subtypes of ischemic stroke included SVO (n = 42), LAA (n = 40), CES (n = 38), and ESUS (n = 20). Of note, in terms of stroke subtypes, the proportion of only the patients with CES who were equol producers was significantly lower than that of patients with HS (HS 48.5% [50/103] vs. SVO 54.8% [23/42], *p* = 0.50; HS vs. LAA 32.5% [14/40], *p* = 0.08; HS vs. CES 28.9% [11/38], *p* = 0.04; HS vs. ESUS 65% [13/29], *p* = 0.18) (Table 2). Further, equol-producing status was significantly associated with a lower prevalence of CES in the logistic analysis adjusted for age, sex, diabetes, dyslipidemia, and hypertension (adjusted odds ratio [aOR]: 0.46, 95% CI: 0.21–0.99, *p* = 0.05) (Figure 1).

Next, cardiac function was examined via TTE. Of 40 equol producers and 53 nonproducers who underwent TTE, no patients was presented with an LVEF of <30%. Echocardiography revealed higher left atrial volume index in the nonproducers than in the producers (46.3 vs. 36.0 mL/m^2^, *p* = 0.06), suggesting the nonproducers tended to have left atrial dysfunction. Other than LAVI, an EF of >50%, LVDd, LVDs, and LAD were not associated with equol-producing status (Table 3).

**Figure 2:**
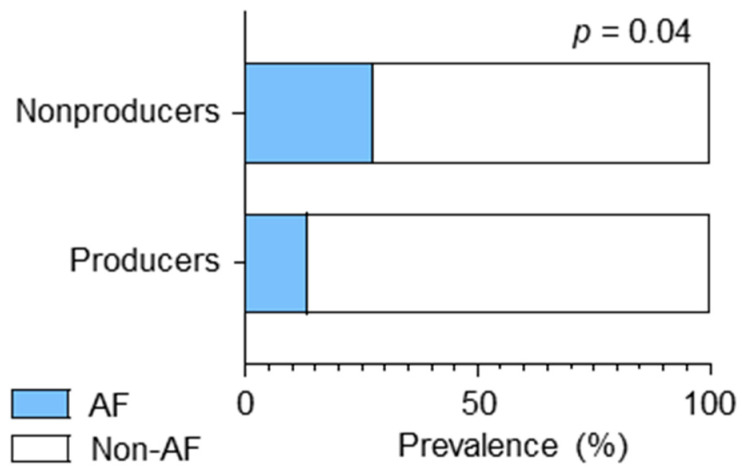
Prevalence of atrial fibrillation (AF). Bar graphs showing the prevalence of AF between the equol nonproducers and producers.

A higher LAVI was associated with AF [40]. Notably, patients with ischemic stroke who were equol nonproducers had a significantly higher prevalemce of AF than those who were producers (27.5% [22/80] vs. 13.3 [8/60], *p* = 0.04) (Figure 2).

### 3.3. Equol-Producing Status as an Independent Predictor of Favorable Functional Outcomes

**Figure 3:**
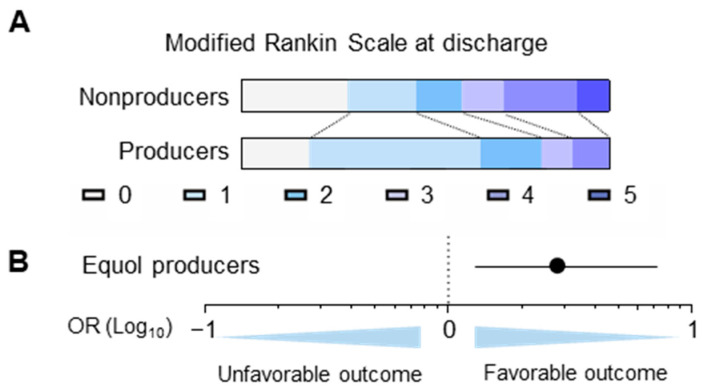
Functional outcome upon discharge after ischemic stroke. (**A**) Bar graphs showing the modified Rankin scale score of the equol nonproducers and producers upon discharge after ischemic stroke. (**B**) Multivariate logistic regression analysis showed that equol production was an independent predictor of favorable functional outcome (the modified Rankin scale score of 0–2).

Equol producers had a significantly lower NIHSS score upon admission of ischemic stroke than nonproducers (3.1 ± 4.1 vs. 7.1 ± 8.3, *p* = 0.01) (Table S3). Additionally, the proportion of producers with favorable functional outcomes upon discharge was significantly higher than that of nonproducers (81.7% vs. 60%, *p* < 0.01) (Figure 3A). The logistic regression analysis adjusted for age, sex, diabetes, dyslipidemia and hypertension was performed on patients with a premorbid mRS score of ≤2. Equol production was an independent indicator of favorable functional outcomes upon discharge (aOR 2.84, 95% CI 1.20–6.75, *p* = 0.018) (Figure 3B).

### 3.4. More Severe SVD in Equol Nonproducers

We assessed the neuroradiological findings of SVD in patients with ischemic stroke (*n* = 137), which is the most common asymptomatic and chronic cerebrovascular disease, via 3-tesla brain MRI. Equol producers had a lower prevalence of overt DSWMH (63.5% vs. 46.6%, *p* = 0.05) and severe SVD (39.7% vs. 57.9%, *p* = 0.04) than nonproducers (Table 4).

After adjusting for age, sex, hypertension, diabetes mellitus, and dyslipidemia, the multivariable logistic regression analysis revealed that equol production was more likely to be inversely associated with a total SVD severity score of ≥2 (aOR 0.50, 95% CI 0.23–1.09, *p* = 0.08).

### 3.5. No Association between Equol-Producing Status and the Development of ICH

HS and patients with ICH were compared via a supplemental analysis. Data on 40 patients with ICH who were admitted to our hospital from September 2019 to October 2021 were collected. The mean age of the patients with ICH was 64.5 ± 13.5 years, and 27 (67.5%) patients were men. The proportion of HS and patients with ICH who were equol producers did not significantly differ (48.5% vs. 40.0%, *p* = 0.36) (Table S4).

## 4. Discussion

This study showed that equol-producing status was significantly associated with a lower prevalence of CES and AF and better functional outcomes after ischemic stroke. Indeed, equol nonproduction may be associated with left atrial dysfunction or atrial cardiomyopathy. Based on the echocardiographic findings in this study, equol nonproducers had a larger LAVI, which indicates left atrial dysfunction. LAVI is a significant risk factor for developing AF [41,42]. An in vitro study revealed that equol can inhibit the activity of voltage-gated potassium channels expressed in HEK293 cell lines. The currents via the Kv1.5, hKv4.3, and hKCNQ1/hKCNE1 channels are significantly inhibited by equol exposure in a dose-dependent manner [43]. The Kv1.5 channel is localized in atrial myocytes and responsible for atrial repolarization [44,45]. This channel is associated with AF and is a potential target for anti-AF drugs [46,47,48]. In addition, the molecular remodeling of hKv4.3 [49] and the genetic mutations or variants of hKCNQ1/hKCNE1 [50,51] are also associated with an increased risk of AF. Hence, equol can be a promising approach to prevent AF development by inhibiting these channels. Further, in this study, the median CHADS2-VASc sore of 30 patients with AF was four (IQR, 3–5), and 28 (94.7%) patients were treated with anticoagulation therapy such as direct oral anticoagulant (DOAC) before admission. The patients in our study were treated appropriately before admission. Therefore, these findings indicated that equol-nonproducing status was strongly associated with CES/AF development. In addition to AF, a high LAVI is an independent risk for heart failure with preserved ejection fraction (HFpEF). Patients with HFpEF, even without AF, are at high risk of stroke [52]. The current study showed that equol nonproducers had preserved EF and increased LAVI. Therefore, equol nonproducers with increased LAVI should be vigilant for the development of symptomatic HFpEF and CES.

The prevalence of CES varies across regions and ethnicities. CES is the most common cause of ischemic stroke in Western countries. However, its prevalence is lower in Asian countries [53]. This discrepancy may be, at least, partially attributed to the fact that only 20–30% of Westerners produce equol in contrast to 50–70% of Asians. This difference is associated with variations in the components of the gut microbiome [15]. That is, more Asians possess equol-producing gut microbiota. Moreover, Asian and Western soy foods have a different isoflavone composition. Fermented soy foods include approximately one-third of the total intake of soy foods. Hence, Asians consume a high proportion of isoflavone aglycons. Aglycons are absorbed faster than glycosides, and they are easier to convert to equol than glycosides, which Westerners predominantly take [54]. Warfarin interacts with food. However, DOACs have fewer interactions with food than warfarin [55]. St. John’s wort, which is a plant commonly used for depression, reduces the serum concentration of DOAC. In addition, herbal products, ginger, and ginkgo biloba may modify the effect of DOAC [55]. However, there was no report showing the association between these materials and equol production. Therefore, equol production after isoflavone intake may lead to differences in the frequency of CES.

Another intriguing finding of the current study was that the equol producers had significantly favorable functional outcomes upon discharge. Several in vivo studies have revealed that equol has neuroprotective effects [19,20,56]. Equol inhibits the upregulation of gp91phox, which is the most important NADPH oxidase responsible for producing free radicals in a rat cerebral ischemia–reperfusion model [19]. In addition, it reduces infarct size via the downregulation of thiobarbituric acid-reactive substances in rats subjected to transient middle cerebral artery occlusion [20]. Consequently, equol emerges as a promising neuroprotective agent for ischemic stroke due to its antioxidant effects.

Equol stimulated endothelial redox signaling and increased nitric oxide (NO) production via NO synthase activation in in vitro and in vivo studies on several vessel types (umbilical vein, aorta, pulmonary artery, and cerebral basilar artery) [57] Previous studies showed that by binding to estrogen receptor β, equol could rapidly stimulate the phosphorylation of the extracellular signal-regulated protein kinase 1/2 and phosphatidylinositol 3-kinase/protein kinase B, leading to the activation of endothelial NO synthase [57]. Moreover, NO production via NOS activation by equol could lead to secondary activation of the guanylate cyclase pathway. Therefore, it can affect treatment and preventive procedures in not only patients with stroke but also those with cardiovascular diseases such as heart failure, pulmonary hypertension, and systemic hypertension.

In addition to symptomatic stroke, equol-producing status may be also associated with SVD, a representative asymptomatic and chronic cerebrovascular disease. The current study showed that equol nonproducing status was associated with severe SVD, including DSWMH. Both AF and HFpEF are associated with an increased risk of developing white matter lesions including DSWMH [58], and cognitive impairment [59,60,61]. A previous study showed that equol-producing status was significantly inversely associated with the volume of white matter lesions in older Japanese individuals with normal cognitive function [22]. A cross-sectional study showed that equol-producing status or intake of equol may have a greater cognitive benefit [62]. Equol could decrease white matter ischemic damage, and cognitive impairment by preventing AF and HFpEF as well as its direct antioxidant and neuroprotective effects.

Apart from CES, there was a partial association between equol nonproducing status and LAA. Previous reports have revealed that equol producers may have less atrial stiffness and thinner intima media thickness of the carotid artery [15]. Similar to the findings of previous studies, a lack of equol production could promote atherosclerotic changes associated with LAA. Contrary, there were no significant associations between equol-producing status and SVO or ESUS. SVO is a phenotype comprising SVD. White matter hyperintensities on brain MRI, a major neuroradiological finding of SVD, are more common and extensive in patients with acute SVO than in those with other stroke subtypes [63]. White matter hyperintensities are caused by several risk factors, which include not only vascular risk factors but also nonvascular nonatheromatous etiologies [64]. Consequently, the influence of equol may be relatively minimal. Except paroxysmal AF, the potential embolic causes of ESUS include heart diseases that have not been clearly suggested as embolic sources, such as aortic stenosis, mitral valvular prolapse, valvular calcification, and arrhythmia, malignancy, paradoxical embolism, and aortogenic embolism [65]. Therefore, the association between equol-producing status and each mechanism associated with ESUS may differ, and several potential causes of ESUS may reduce the impact of equal-producing status.

Our study had several limitations. First, information about daily diet, physical activity and nutrition was not assessed. Second, the maximum period from the onset of stroke to blood sample collection was six months. Therefore, patients might have changed their food habits within that timeframe. However, a recent study revealed that the equol-producing status remained stable for at least one year [66,67]. Therefore, any potential influences from dietary changes during this period should be negligible. Third, due to the retrospective observational nature of the study, selection biases could have existed, and a small sample size could have been included upon enrollment of patients with acute stroke in this study. These phenomena could be attributed to the following: first, the healthy subjects and patients with acute stroke were not selected from the same cohort; second, our study included strict exclusion criteria, as shown in the Section 2.3; Patients with stroke. Third, this study only included Japanese individuals. The small sample volume and selection bias could cause type 1 and type 2 errors. Fourth, there might be unmeasured cofounding factors for stroke development. Thus, a prospective study involving a larger sample size should be performed. However, the strict exclusion criteria could lead to the identification of the associations of equol nonproducing status with CES and AF.

## 5. Conclusions

Equol is a promising candidate for interventions aiming to reduce the risk of CES and AF, and improve neurological prognosis after ischemic stroke.

## Figures and Tables

**Table 1:** Baseline characteristics of the healthy subjects and patients with acute ischemic stroke.

	Healthy Subjects	Patients with Ischemic Stroke	*p* Value
	*n* = 103	*n* = 140	
Age (years)	81.3 ± 3.3	72.2 ± 12.1	<0.01
Male sex, n (%)	52 (50.5)	102 (72.9)	<0.01
BMI (kg/m^2^)	22.5 ± 3.1	23.1 ± 3.8	0.31
Medical history			
Hypertension, n (%)	56 (54.4)	110 (78.6)	<0.01
Dyslipidemia, n (%)	58 (56.3)	77 (55)	0.84
Diabetes mellitus, n (%)	13 (12.6)	29 (20.7)	0.1
Coronary artery disease, n (%)	0	8 (5.7)	–
Atrial fibrillation, n (%)	3 (2.9)	30 (21.4)	<0.01
Laboratory data			
WBC count (×10^3^/μL)	5.6 ± 1.4	6.9 ± 2.1	<0.01
Platelet count (×10^3^/μL)	203.2 ± 53.2	214.7 ± 62.3	0.24
Albumin level (g/dL)	4.1 ± 0.3	4.0 ± 0.5	0.09
ALT level (U/L)	16.3 ± 9.2	19.8 ± 11.5	<0.01
AST level (U/L)	23.2 ± 7.0	24.1 ± 7.5	0.22
Creatinine level (mg/dL)	0.8 ± 0.2	1.0 ± 1.8	<0.01
HDL-C level (mg/dL)	58.1 ± 14.0	52.1 ± 14.9	<0.01
LDL-C level (mg/dL)	120.0 ± 23.5	117.3 ± 38.4	0.33
TG level (mg/dL)	105.1 ± 56.9	128.9 ± 73.7	0.01

Abbreviations: BMI, body mass index; WBC, white blood cell; ALT, alanine aminotransferase; AST, aspartate aminotransferase; HDL-C, high-density lipoprotein cholesterol; LDL-C, low-density lipoprotein cholesterol; TG triglyceride.

**Table 2:** Baseline characteristics of patients with various stroke subtypes.

	Patients with SVO *n* = 42	Patients with LAA *n* = 40	Patients with CES *n* = 38	Patients with ESUS *n* = 20
Age (years), mean ± SD	70.9 ± 11.5	68.3 ± 12.0	78.1 ± 9.0	71.6 ± 15.1
Male sex, n (%)	34 (81.0)	33 (82.5)	22 (57.9)	13 (65.0)
BMI (kg/m^2^)	23.5 ± 3.7	23.4 ± 3.2	22.3 ± 4.6	23.4 ± 3.8
Medical history				
Hypertension, n (%)	31 (73.8)	36 (90.0)	27 (71.1)	16 (80.0)
Dyslipidemia, n (%)	22 (52.4)	28 (70.0)	14 (36.8)	13 (65.0)
Diabetes mellitus, n (%)	7 (16.7)	14 (35.0)	5 (13.2)	3 (15.0)
CAD, n (%)	1 (2.4)	2 (5.0)	4 (10.5)	1 (5.0)
CHA_2_DS_2_-VASc score, median [IQR]	2.5 [2–4]	2.5 [2–4]	3.5 [3–5]	3.5 [2.5–4]
LDL-C-lowering drugs, n (%)	29 (69.0)	38 (95.0)	18 (47.4)	16 (80.0)
Anticoagulants, n (%)	3 (7.1)	4 (10.0)	36 (94.7)	2 (10.0)
NIHSS score upon admission, mean ± SD	2.0 ± 2.4	3.7 ± 4.4	11.7 ± 9.4	3.8 ± 5.2
Premorbid mRS score, mean ± SD	0.5 ± 1.0	0.2 ± 0.6	1.0 ± 1.6	0.5 ± 1.3
Equol producers, n (%)	23 (54.8)	13 (32.5)	11 (29.0)	13 (65.0)

Abbreviations: SD, standard deviation; IQR, interquartile range; SVO, small-vessel occlusion; LAA, large-artery atherosclerosis; CES, cardioembolic stroke; ESUS, embolic stroke of undetermined source; BMI, body mass index; CAD, coronary artery disease; LDL-C, low-density lipoprotein cholesterol; NIHSS, National Institutes of Health Stroke Scale; mRS, modified Rankin Scale.

**Table 3:** Echocardiographic findings of the equol producers and nonproducers.

	Equol Nonproducers	Equol Producers	*p* Value
LVEF < 50%	5/50 (10.0%)	5/38 (13.2%)	0.74
LVDd (mm)	46.4 ± 5.2	47.8 ± 5.5	0.53
LVDs (mm)	30.9 ± 5.0	31.8 ± 6.2	0.62
LAD (mm)	39.5 ± 6.4	37.5 ± 6.5	0.13
LAVI (mL/m^2^)	46.3 ± 23.8	36.0 ± 11.6	0.06

Abbreviations: LVEF, left ventricular ejection fraction; LVDd, left ventricular diastolic diameter; LVDs, left ventricular systolic diameter; LAD, left atrial diameter; LAVI, left atrial volume index.

**Table 4:** Neuroradiological features of small-vessel disease (SVD) between the equol producers and nonproducers.

	Equol Nonproducers	Equol Producers	*p*
PVH (grade 3), n (%)	13/74 (17.6)	7/58 (12.1)	0.38
DSWMH (grade ≥ 2), n (%)	47/74 (63.5)	27/58 (46.6)	0.05
Lacunes ≥ 1, n (%)	39/74 (52.7)	26/58 (44.8)	0.37
CMBs ≥ 1, n (%)	27/77 (35.1)	20/60 (33.3)	0.83
Total SVD severity ≥ 2, n (%)	42/73 (57.5)	23/58 (39.7)	0.04

Abbreviation: PVH, periventricular hyperintensities; DSWMH, deep and subcortical white matter hyperintensities; CMBs, cerebral microbleeds.

## Data Availability

The data in this study are available from the corresponding author upon reasonable request.

## Supplementary Materials

The following supporting information can be downloaded at https://www.mdpi.com/article/10.3390/nu16193377/s1, Table S1: Prevalence of patients with dyslipidemia and LDL-C lowering drug among patients with stroke; Table S2: Baseline characteristics of healthy subjects and patients with acute ischemic stroke who were equol producers and nonproducers; Table S3: Baseline characteristics of patients with ischemic stroke who were equol producers and nonproducers; Table S4: Baseline characteristics of healthy subjects and patients with intracranial hemorrhage.
