# Supplementary material for: Equol Nonproducing Status as an Independent Risk Factor for Acute Cardioembolic Stroke and Poor Functional Outcome
Source: Nutrients. 2024 Oct 4;16(19):3377. doi: 10.3390/nu16193377 (PMC11479244; doi:10.3390/nu16193377)
Supplement: Supplementary file 1 [file nutrients-16-03377-s001.zip › nutrients-3217154-supplementary.pdf]

Supplementary Material

Equol Nonproducing Status as an Independent Risk Factor for Acute Cardioembolic Stroke and Poor Functional Outcome

Kotaro Noda, MD; Yorito Hattori, MD, PhD; Hiroaki Murata, MD; Yoshihiro Kokubo, MD, PhD; Aya Higashiyama, MD, PhD; Masafumi Ihara, MD, PhD

Correspondence:
Yorito Hattori: yoh2019@ncvc.go.jp

Supplementary Tables

Table S1. Prevalence of patients with dyslipidemia and low-density lipoprotein cholesterol (LDL-C) lowering drug among patients with stroke.

	**Patients treated with LDL-C lowering drug n = 101**	**Patients not treated with LDL-C lowering drug n = 39**
Dyslipidemia, n (%)	71 (70.3)	6 (15.4)

Table S2. Baseline characteristics of healthy subjects and patients with acute ischemic stroke who were equol producers and nonproducers.

	**Equol nonproducers n = 133**	**Equol producers n = 110**	***p* value**
Age (years)	76.1 ± 10.6	76.0 ± 10.4	0.98
Male sex, n (%)	76 (57.1%)	78 (70.9%)	0.03
BMI (kg/m^2)	23.2 ± 4.1	22.5 ± 2.8	0.30
Medical history			
Hypertension, n (%)	93 (69.9%)	73 (66.4%)	0.55
Dyslipidemia, n (%)	72 (54.1%)	63 (57.3%)	0.62
Diabetes mellitus, n (%)	24 (18.0%)	18 (16.4%)	0.73
Coronary artery disease, n (%)	3 (2.3%)	5 (4.5%)	0.32
Atrial fibrillation, n (%)	23 (17.3%)	10 (9.1%)	0.06
Laboratory data			
WBC count (× 10^3/μL)	6.4 ± 2.0	6.2 ± 1.9	0.19
Platelet count (× 10^3/ μL)	215.9 ± 66.3	217.0 ± 156.8	0.26
Albumin level (g/dL)	4.1 ±0.4	4.1 ± 0.3	0.99
ALT level (U/L)	18.7 ± 11.7	17.9 ± 9.5	0.83
AST level (U/L)	23.5 ± 7.2	24.0 ± 7.4	0.56
Creatinine level (mg/dL)	1.0 ± 0.7	1.0 ± 0.7	0.35
HDL-C level (mg/dL)	54.1 ± 15.2	55.5 ± 14.3	0.42
LDL-C level (mg/dL)	117.8 ± 32.9	117.3 ± 32.8	0.77
TG level (mg/dL)	119.5 ± 67.4	117.7 ± 68.8	0.76

Abbreviations: BMI—body mass index; NIHSS—National Institutes of Health Stroke Scale; mRS—modified Rankin Scale; WBC—white blood cell; ALT—alanine aminotransferase; AST—aspartate aminotransferase; HDL-C—high-density lipoprotein cholesterol; LDL-C—low-density lipoprotein cholesterol; TG—triglycerides.

Table S3. Baseline characteristics of patients with ischemic stroke who were equol producers and nonproducers.

	**Equol nonproducers n = 80**	**Equol producers n = 60**	***p* value**
Age (years)	73.1 ± 12.5	71.1 ± 11.6	0.30
Male sex, n (%)	50 (62.5)	52 (86.7)	< 0.01
BMI (kg/m^2)	23.3 ± 4.5	22.9 ± 2.8	0.91
Medical history			
Hypertension, n (%)	63 (78.8)	47 (78.3)	0.95
Dyslipidemia, n (%)	43 (53.8)	34 (56.7)	0.73
Diabetes mellitus, n (%)	16 (20.0)	13 (21.7)	0.81
Coronary artery disease, n (%)	3 (3.8)	5 (8.3)	0.29
Atrial fibrillation, n (%)	22 (27.5)	8 (13.3)	0.04
NIHSS score upon admission	7.1 ± 8.3	3.1 ± 4.1	0.01
mRS score upon admission	0.8 ± 1.4	0.3 ± 0.8	0.03
Favorable outcome, n (%)	48 (60.0%)	49 (81.7%)	< 0.01
Laboratory data			
WBC count (× 10^3/μL)	6.9 ± 2.2	6.8 ± 2.0	0.92
Platelet count (× 10^3/μL)	221.6 ± 71.5	205.5 ± 46.4	0.27
Albumin level (g/dL)	4.0 ± 0.5	4.1 ± 0.3	0.88
ALT level (U/L)	19.7 ± 13.6	20.0 ± 8.1	0.11
AST level (U/L)	24.0 ± 7.9	24.3 ± 7.0	0.80
Creatinine level (mg/dL)	1.0 ± 0.8	1.1 ± 0.9	0.14
HDL-C level (mg/dL)	52.0 ± 15.5	52.3 ± 14.2	0.87
LDL-C level (mg/dL)	117.8 ± 37.7	116.6 ± 39.6	0.73
TG level (mg/dL)	126.8 ± 78.6	131.9 ± 66.8	0.35
BNP level (pg/mL)	194.6 ± 344.4	135.9 ± 341.2	0.17

Abbreviations: BMI—body mass index; NIHSS—National Institutes of Health Stroke Scale; mRS—modified Rankin Scale; WBC—white blood cell; ALT—alanine aminotransferase; AST—aspartate aminotransferase; HDL-C—high-density lipoprotein cholesterol; LDL-C—low-density lipoprotein cholesterol; TG—triglycerides; BNP—brain natriuretic peptide.

Table S4. Baseline characteristics of healthy subjects and patients with intracranial hemorrhage.

	**Healthy subjects** **n = 103**	**Patients with intracranial hemorrhage** **n = 40**	***p* value**
Age (years)	81.3 ± 3.3	64.5 ± 13.5	< 0.01
Male sex, n (%)	52 (50.5%)	27 (67.5%)	0.07
BMI (kg/m^2)	22.5 ± 3.1	24.2 ± 5.1	0.2
Medical history			
Hypertension, n (%)	56 (54.4%)	100 (100.0%)	-
Dyslipidemia, n (%)	58 (56.3%)	12 (30.0%)	0.01
Diabetes mellitus, n (%)	13 (12.6%)	5 (12.5%)	0.98
Coronary artery disease, n (%)	0	1 (2.5%)	-
Atrial fibrillation, n (%)	3 (2.9%)	0	-
Equol producer, n (%)	50 (48.5%)	16 (40.0%)	0.36
Laboratory data			
WBC count (× 10^3/μL)	5.6 ± 1.4	8.3 ± 2.8	< 0.01
Platelet count (× 10^3/μL)	203.2 ± 53.2	235.9 ± 77.4	< 0.01
Albumin level (g/dL)	4.1 ± 0.3	4.1 ± 0.5	0.65
ALT level (U/L)	16.3 ± 9.2	23.0 ± 15.2	< 0.01
AST level (U/L)	23.1 ± 7.0	25.9 ± 9.0	0.06
Creatinine level (mg/dL)	0.8 ± 0.2	1.0 ± 0.8	0.33
HDL-C level (mg/dL)	58.1 ± 14.0	54.5 ± 15.3	0.03
LDL-C level (mg/dL)	120.0 ± 23.5	117.6 ± 36.7	0.34
TG level (mg/dL)	105.1 ± 56.9	125.0 ± 85.9	0.23

Abbreviations: BMI—body mass index; NIHSS—National Institutes of Health Stroke Scale; mRS—modified Rankin Scale; WBC—white blood cell; ALT—alanine aminotransferase; AST—aspartate aminotransferase; HDL-C—high-density lipoprotein cholesterol; LDL-C—low-density lipoprotein cholesterol; TG—riglycerides.
